# The Safety of EXPAREL ® (Bupivacaine Liposome Injectable Suspension) Administered by Peripheral Nerve Block in Rabbits and Dogs

**DOI:** 10.1155/2012/962101

**Published:** 2012-01-17

**Authors:** Brigitte M. Richard, Paul Newton, Laura R. Ott, Dean Haan, Abram N. Brubaker, Phaedra I. Cole, Paul E. Ross, Marlon C. Rebelatto, Keith G. Nelson

**Affiliations:** ^1^Clinical Research and Drug Safety Assessment, Pacira Pharmaceuticals, Inc., 10450 Science Center Drive, San Diego, CA 92121, USA; ^2^MPI Research Laboratories, North Main Street, Mattawan, MI 49071, USA

## Abstract

A sustained-release DepoFoam injection formulation of bupivacaine (EXPAREL, 15 mg/mL) is currently being investigated for postsurgical analgesia via peripheral nerve block (PNB). Single-dose toxicology studies of EXPAREL (9, 18, and 30 mg/kg), bupivacaine solution (Bsol, 9 mg/kg), and saline injected around the brachial plexus nerve bundle were performed in rabbits and dogs. The endpoints included clinical pathology, pharmacokinetics, and histopathology evaluation on Day 3 and Day 15 (2/sex/group/period). EXPAREL resulted in a nearly 4-fold lower *C*_max_ versus Bsol at the same dose. EXPAREL was well tolerated at doses up to 30 mg/kg. The only EXPAREL-related effect seen was minimal to mild granulomatous inflammation of adipose tissue around nerve roots (8 of 24 rabbits and 7 of 24 dogs) in the brachial plexus sites. The results indicate that EXPAREL was well tolerated in these models and did not produce nerve damage after PNB in rabbits and dogs.

## 1. Introduction

Bupivacaine is a local anesthetic/analgesic widely used in the perioperative and postsurgical settings. Bupivacaine solutions have been used for many years by multiple routes for the relief of postoperative pain [1]. With technology now allowing for directly visualizing a peripheral nerve prior to injection, perineural nerve block, including brachial plexus nerve block, has become increasingly popular.

The brachial plexus is a large, complex bundle of nerves (arising from the nerve roots C5-T1). A single injection of local anesthetic around the brachial plexus nerve bundle results in block of arm tissue innervated by several peripheral nerves. Several approaches to the brachial plexus blockade have been described (i.e., the axillary, infraclavicular, supraclavicular, and interscalene) and each have advantages in certain situations. Stabilization of the needle for catheter insertion after brachial plexus blockade is localized and is a challenging aspect of this technique [2].

Brachial plexus blockade may require dispersion of a relatively large volume of bupivacaine in solution to accomplish blockade of the entire plexus. Complications may include infection, hematoma, vascular puncture, toxicity, injury, and total spinal anesthesia [3]. After performing the block procedure, peripheral nerves may be damaged from prolonged contact with concentrated formulations [4, 5]. From a systemic standpoint, high doses of bupivacaine may be associated with a wide range of systemic complications, such as central nervous and cardiovascular effects [6].

A formulation of bupivacaine that provides prolonged release of the active ingredient after a single administration would simplify pain management in the postoperative period and eliminate the undesired peak plasma concentrations as a result of excessively high concentrations and reduce the risk of local and systemic reactions [7].

EXPAREL (bupivacaine liposome injectable suspension) is a sterile suspension of multivesicular liposomes using proprietary DepoFoam formulation technology to release bupivacaine over several days. EXPAREL, the proposed proprietary name, was designed to provide prolonged analgesia for 72 hours after wound infiltration in patients [8].

Although different liposomal formulations have been administered to humans without toxicity [9], the in vivo tolerability of liposomes continues to be investigated. Our goal was to evaluate the potential local and systemic toxicity of EXPAREL after a bolus injection into the brachial plexus (i.e., a large, complex bundle of nerves in the shoulder). Specifically, the study was designed to assess whether EXPAREL did not produce nerve damage in the setting of peripheral nerve block by comparison with unencapsulated bupivacaine or saline control.

## 2. Materials and Methods

### 2.1. Materials

#### 2.1.1. Description of DepoFoam Technology

The DepoFoam drug-delivery system is a proprietary, injectable technology that provides a sustained release of therapeutic compounds. The DepoFoam system consists of multivesicular liposomes composed of hundreds to thousands of chambers per particle, resembling a “honeycomb-like” matrix [10].

Such particles are to be distinguished from multilamellar vesicles, also known as a multilamellar liposome, which contain multiple concentric membranes within each liposome particle [11, 12].

The DepoFoam particle components are naturally occurring or synthetic analogues of common lipids, including phospholipids (e.g., dierucoylphosphatidylcholine and dipalmitoylphosphatidylglycerol), cholesterol, and triglycerides (e.g., triolein and tricaprylin). The particles typically consist of >97% water (with dissolved drug) and 1% to 3% lipids, and are expected to be fully biodegradable. The DepoFoam particles are typically suspended in isotonic solutions containing sodium chloride 0.9% in water for injection. The DepoFoam drug-delivery system is already used in two marketed products, DepoDur and DepoCyt, which are produced by Pacira Pharmaceuticals, Inc.

#### 2.1.2. Description of DepoFoam Bupivacaine

DepoFoam bupivacaine (bupivacaine liposome injectable suspension), was supplied by Pacira Pharmaceuticals, Inc., San Diego, California, USA. This formulation was previously designated SKY0402.

The manufacture of DepoFoam particles has been previously described [12]. Briefly, the process involves a double emulsification process where the bupivacaine is added as part of the initial emulsification process. The amount of unencapsulated bupivacaine is controlled as part of the process and is generally less than 10%. In DepoFoam Bupivacaine, one of the specific lipids in the final formulation is dierucoylphosphatidylcholine, EXPAREL was initially formulated at two different dose concentrations (15 and 25 mg/mL in 0.9% saline, expressed as anhydrous bupivacaine HCl equivalent). The 15-mg/mL formulation is intended for commercial use. The 15 mg/mL of bupivacaine is the bupivacaine salt HCl; it is chemically equivalent to 13.3 mg/mL bupivacaine free base. The 25-mg/mL formulation is a concentrated version and was intended to increase exposure of local tissues to relatively higher concentrations of both the active drug and DepoFoam matrix.

#### 2.1.3. Reference Product

Sensorcaine-MPF (methyl paraben free; bupivacaine HCl injection, USP) 0.75% bupivacaine solution, is manufactured by AstraZeneca, Wilmington, Delaware, USA.

#### 2.1.4. Control Article

Saline (0.9% sodium chloride injection, USP) is manufactured by Abbott Laboratories, North Chicago, lllinois, USA.

#### 2.1.5. Animals

New Zealand White rabbits and beagle dogs were ordered from Covance Research Products, Philadelphia, Pennsylvania, and Kalamazoo, Michigan, USA, respectively. The animals were 5 and half months (rabbit) and 5 to 6 months (dog) of age on arrival.

A total of 40 rabbits (20 males and 20 females) weighing 2.6 to 3.7 kg and 40 dogs (20 males and 20 females) weighing 6.2 to 9.7 kg, were used. Individual body weights were within 20% of the mean body weight for each gender.

### 2.2. Methods

#### 2.2.1. Study Protocols

These pivotal studies were conducted according to International Conference on Harmonisation guidelines in accordance with Good Laboratory Practices principles [13] as set forth by the United States Food and Drug Administration 21 CFR Part 58 and in accordance with single-dose acute toxicity testing for pharmaceuticals [14]. All protocols were reviewed and approved by the Institutional Animal Care and Use Committee of MPI Research for compliance with regulations prior to study initiation.

The brachial nerve block procedure was performed in an MPI Research surgical suite using aseptic technique. General anesthesia was induced and maintained with isoflurane to effect delivered in oxygen through a facemask. Each dose was administered by nerve block once on Day 1. A 22 g 3.5 inch needle was used to perform a brachial plexus nerve block in the left thoracic limb [15]. After placement the needle was slowly withdrawn while each treatment was injected. The animals were closely monitored during anesthetic recovery for physiological disturbances including cardiovascular/respiratory depression, hypothermia, and excessive bleeding from the injection site.

Using a standard, by weight, block randomization procedure, 20 males and 20 females were assigned to five groups in each study. Each group consisted of 4 males and 4 females. Groups 1, 2, and 3 received EXPAREL 9, 18, and 25 mg/kg (or 30 mg/kg), respectively. Group 4 received bupivacaine solution 9 mg/kg, Group 5 received saline.

In rabbits, EXPAREL was administered at a dose volume of 0.6, 1.2, and 1.2 mL/kg, respectively. In dogs, EXPAREL was administered at a dose volume of 0.6, 1.2, and 1.0 mL/kg, respectively. (The dogs intended for the 30 mg/kg group were erroneously administered only 1.0 mL/kg (25 mg/mL) resulting in a dose level of 25 mg/kg.) Bupivacaine solution and saline control was administered at a dose volume of 1.2 mL/kg in each species.

Following dose administration, animals were observed without further treatment. Four animals (2 males and 2 females) in each group were sacrificed on Day 3, and the remainder (designated as recovery animals, 2 males and 2 females) were observed for an additional two weeks until Day 15 sacrifice.

Endpoints included physical examinations, clinical signs, clinical pathology, hematology, organ weight, and histopathology of a standard list of tissues (including injection sites) on Day 3 and Day 15, as well as pharmacokinetics (PK) on Day 1 through 96 hours postdosing.

#### 2.2.2. Pharmacokinetic Data Analysis

Plasma bupivacaine concentrations were measured using a validated LC/MS/MS assay in rabbit and dog K_3_EDTA plasma in the concentrations ranging from 10.0 to 10,000 ng/mL. The lower quantitation limit was 10 ng/mL.

The PK parameters were evaluated by a noncompartmental model using WinNonlin, version 5.0 (Pharsight Corp., Mountain View, California). The PK parameters were maximum plasma concentration (*C*_max⁡_), time at which the *C*_max⁡_ occurred (*t*_max⁡_), and area under the plasma concentration-time data from time 0 to selected time point (*t*) (AUC_0-*t *_). The appropriate group mean values and standard deviation were calculated from the individual data (combined sexes). Statistical results of pairwise comparisons between EXPAREL and bupivacaine solution groups were reported at the 0.05 significance levels using Student's two-tailed *t*-test.

#### 2.2.3. Tissue Processing and Microscopic Evaluation

All animals had a complete necropsy examination. Organ weights were recorded for the following organs prior to fixation: adrenal glands, brain, heart, kidneys, liver, lungs (with bronchi), ovaries, spleen, testes, and thyroid. Paired organs were weighed together.

A selection of routine tissues (approximately 70) including gross lesions, injection sites and surgical wound tissues were collected at necropsy from 2 males and 2 females per group sacrificed on Day 3 and remaining 2 males and 2 females on Day 15 (recovery group). Tissues were trimmed, embedded, sectioned, and hematoxylin- and eosin-stained using standard procedures. All pathology slides were prepared by MPI Research Laboratories. The severity of histological findings was graded on a scale of one to four with 0 = none, 1 = minimal, 2 = mild, 3 = moderate, and 4 = severe. All protocol-specified tissues were examined, and grading/interpretations of findings were made by a pathologist certified by the American College of Veterinary Pathology.

The nerve plexus site was excised, and histopathological preparations were prepared across the complete site. Nerve plexus sites examined microscopically at the three sampling sites with as much nerve and connective tissue as possible (proximal, middle, and distal to the injection sites).

All changes in the skin and underlying muscle tissues and other organs were recorded.

Neurotoxicity was assessed primarily on a histopathological level using light microscopic evaluation of hematoxylin- and eosin-stained injection sites. Any neural changes observed at the injection sites would typically be listed as separate findings (such as degeneration or inflammation).

## 3. Results

### 3.1. Toxicology Results

In both rabbits and dogs, a single-dose administration of EXPAREL was well tolerated even at a large dose and concentration (up to 30 mg/kg, 25 mg/mL). There were no discernable EXPAREL-related effects on hematology, clinical chemistry, or urinalysis parameters (data not shown). Few sporadic changes were noted at termination or recovery, but these effects were considered not toxicologically relevant, may be the results of biological variability, and were not considered treatment-related.

Microscopic findings at the brachial plexus sites in male and female rabbits and dogs (combined sexes) are shown in Tables 1 and 2. Microscopic findings on Day 3 consisted of granulomatous inflammation and hemorrhage; females also had minimal subacute inflammation. On Day 15, brachial plexus lesions included granulomatous inflammation and hemorrhage; females also had minimal fibrosis; males also had subacute inflammation and mineralization.

The only EXPAREL-related effect seen was minimal to mild granulomatous inflammation of adipose tissue around nerve roots (8 of 24 rabbits and 7 of 24 dogs) in brachial plexus sites. Granulomatous inflammation was present in 4/12 rabbits on Day 3 or Day 15, and in only 1/12 dog (Day 3) and 6/12 dogs (Day 15). Apart from granulomatous inflammation observed at the injection sites, there was no overall incidence or severity of lesions in the brachial plexus between animals receiving EXPAREL and the saline control or Bupivacaine solution groups. All other microscopic findings were considered incidental and unrelated to EXPAREL.

**Figure 1:**
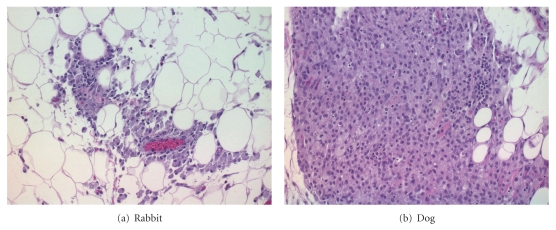
Injection site findings (Day 3) in a female rabbit (a) or dog (b) of the EXPAREL 18 mg/kg (a) and 25 mg/kg (b) showing granulomatous inflammation of adipose tissue. H&E 20X.

This change was characterized by aggregates of macrophages with abundant vacuolated cytoplasm (Figures 1(a) and 1(b)). With the low incidence and severity of these effects, this reaction was considered a normal response to the liposomes and not adverse. There was no other difference in the incidence or severity of lesions between groups.

### 3.2. Pharmacokinetic Results

**Figure 2:**
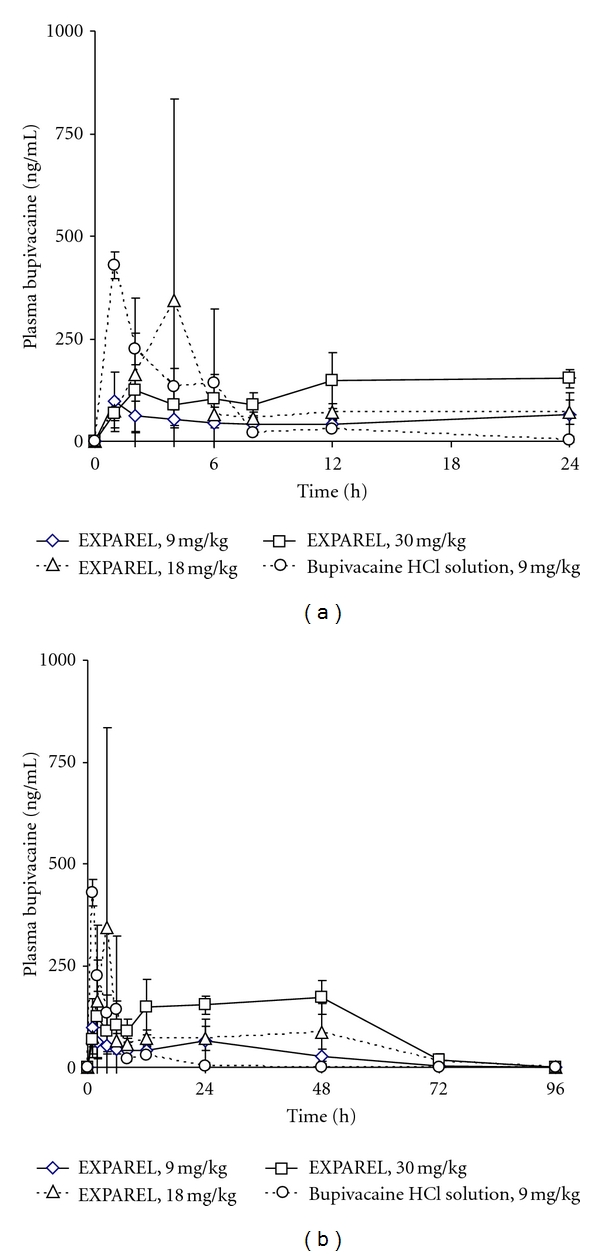
Mean pharmacokinetic profile of EXPAREL in rabbits from 0–24 hours (a) and 0–96 hours (b).

In rabbits, *C*_max⁡_ values were dose dependent, averaging 106 ± 67.9,  363 ± 478  and 205 ± 60.4 ng/mL for the three EXPAREL dose levels (9, 18, and 30 mg/kg, resp.) (Figure 2(a)). As a result of the relatively flat nature of the concentration-time profile over the first 48 hours, the mean time to maximum plasma concentration, *t*_max⁡_, varied considerably: 10.3 ± 10.3, 20.0 ± 20.1, and 36.5 ± 23.0 hours for the three doses (Figure 2(b)). The AUC_0–96 h_ values determined for each of the three doses were 2700 ± 781,5540 ± 2520, and 9370 ± 1170 ng·h/mL indicating dose proportionality.

 These results can be compared with the PK values found for the bupivacaine solution administered at the lowest dose, 9 mg/kg. The plasma bupivacaine concentration peaked quickly and fell below the limit of detection by 48 hours. The *C*_max⁡_, *t*_max⁡_, and AUC_0–96 h_ were 433 ± 26.2 ng/mL, 2.25 ± 2.50 hours and 1670 ± 249 ng·h/mL, respectively.

**Figure 3:**
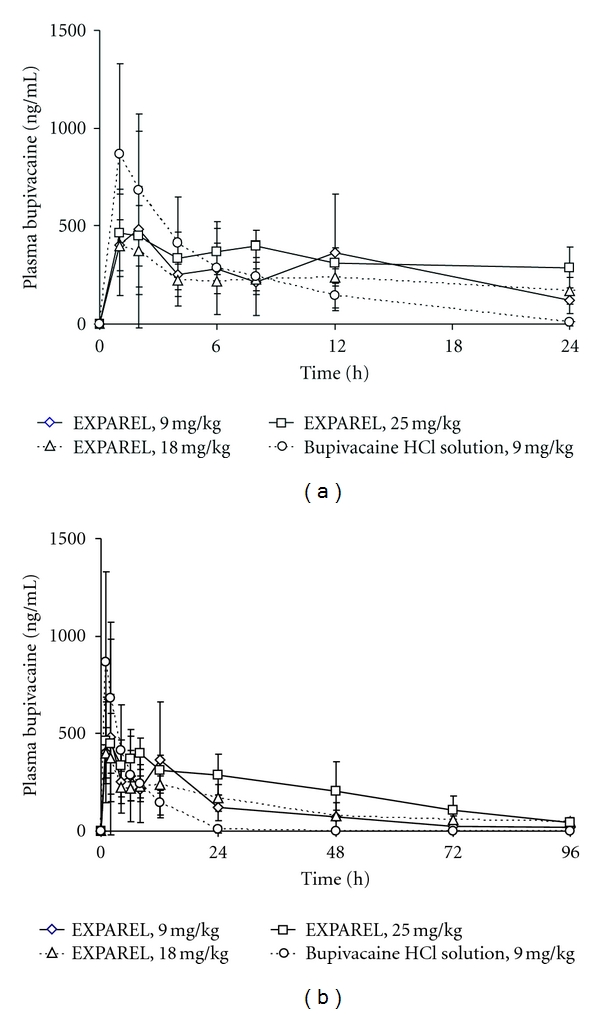
Mean pharmacokinetic profile of EXPAREL in dogs from 0–24 hours (a) and 0–96 hours (b).

In dogs receiving bupivacaine solution (9 mg/kg), plasma bupivacaine concentrations peaked quickly (*t*_max⁡_ of 1.00 ± 0.00 hour, *C*_max⁡_ of 1490 ± 131 ng/mL) and declined rapidly thereafter (Figure 3(a)). Half-life was estimated to be 5.92 ± 2.51 hours. The AUC_0–96 h_ value was 6100 ± 1520 ng·h/mL.

 Detectable plasma bupivacaine concentrations were observed in most animals with the EXPAREL formulation (9 mg/kg) over the entire 96-hour study period (Figure 3(b)). The bupivacaine plasma concentrations declined slowly over time, and the *C*_max⁡_ values were dose dependent, averaging 402 ± 513, 715 ± 747, and 1130 ± 604 ng/mL for the three dose levels (9, 18, and 25 mg/kg, resp.). The corresponding AUC_0–96 h_ values were 7460 ± 1370, 18200 ± 8640, and 22600 ± 13700 ng·h/mL. When comparing the results in both species, the peak concentrations were reduced and elevated plasma drug concentrations were maintained for longer periods with EXPAREL compared to bupivacaine solution at the same dose (9 mg/kg) in both species. Occasional and expected differences in individual PK parameters were present although not observed across all groups. Particularly, due to the known variability in the absorption of bupivacaine, *C*_max⁡_ was found to be higher in one of the three animals receiving the intermediate formulations (18 mg/kg). In this dose group, the individual plasma *C*_max⁡_ values were 1080, 162, 108, and 103 ng/mL achieved at 4, 48, 24, and 4 hours and 1790, 648, 239, and 181 ng/mL at 1, 24, 24, and 2 hours, in rabbits and dogs, respectively.

The attenuation of *C*_max⁡_ with EXPAREL (9 mg/kg) was 4.1 and 3.7 fold in both rabbits and dogs (combined sexes), respectively. The difference was statistically significant compared to bupivacaine solution at the same dose (*P* < 0.05). 

The AUC_0–24 h_ was statistically significantly lower in dogs with EXPAREL compared to bupivacaine solution (2860 ± 1400 versus 6020 ± 1380 ng·h/mL, 2-fold difference, *P* < 0.05), while the corresponding values in rabbits were not significantly different (1230 ± 536 versus 1620 ± 288 ng·h/mL). 

The AUC_0–96 h_ was statistically significantly greater in rabbits with EXPAREL compared to bupivacaine solution at the same dose (9 mg/kg,  2700 ± 781  versus 1670 ± 249 ng·h/mL (1.6 fold difference *P* < 0.05), whereas the corresponding values in dogs were not significantly different (7460 ± 1370 versus 6100 ± 1520 ng·h/mL) in dogs.

## 4. Discussion

The ultimate goal is to design a liposomal bupivacaine formulation which would produce maximum prolongation of analgesia without causing local or systemic toxicity. In our studies, we evaluated the local and systemic toxicity produced by EXPAREL in comparison with bupivacaine solution and saline after a single bolus injection around the brachial plexus nerve bundle. Since the local and systemic toxicity of bupivacaine solution is well known, our experiment focuses on showing that EXPAREL did not cause overt irritation or local tissue damage even when used at high dose or concentration.

This is the first reported toxicology evaluation of EXPAREL using brachial plexus block. We used both a clinical concentration of 15 mg/mL and a higher concentration of 25 mg/mL for a total dosing up to 30 mg/kg to demonstrate a wider safety margin for both concentration and total dosing of bupivacaine and lipid components. Brachial plexus blockade was selected as the large network of nerve fibers which distributes the innervation of the upper extremity is clinically relevant. 

Neurological damage is a well-recognized side effect of local analgesics applied in high concentrations close to neuronal structures such as peripheral nerves, nerve plexuses, or the spinal cord [16]. Local analgesics do not cause any direct nerve damage unless they are injected intraneurally or given in higher concentrations than that which is commercially available. Several different laboratory models have proven that all local analgesics can be neurotoxic but that lidocaine and tetracaine are potentially more neurotoxic than bupivacaine [17].

The pathogenesis of local analgesics-induced local tissue toxicity is poorly understood. There appears to be a relationship between concentration and neurotoxicity. In 1985, Ready et al. [18] evaluated the neurotoxic effects of single injections of local analgesics in rabbits. They reported that spinal cord histopathology remained normal and that persistent neurologic deficits were not seen with clinically used concentrations of tetracaine, lidocaine, bupivacaine, or chloroprocaine. However, histopathologic changes and neurologic deficits did occur with higher concentrations of tetracaine (1%, up to 8%) and lidocaine (8%, up to 32%). It was found that high concentrations of lidocaine (and tetracaine) caused neural injury. Notably, in this model, extensive neurologic impairment was not necessarily accompanied by equally extensive lesions in the spinal cord and nerve roots, thus demonstrating the need for multiple models to fully assess neurotoxicity. Particularly, the highest concentration of bupivacaine (3.3%) was not consistently associated with comparable neural damage.

Peripheral nerve injury is a rare complication of regional anesthesia. The pathogenesis of local analgesics-induced local tissue toxicity is poorly understood. The mechanism of this enhanced toxicity remains to be established, but it may be related to an effect of diverse vasoconstriction on anesthetic exposure [19]. Ischemia is one of the possible causative mechanisms which may result from changes in peripheral blood flow caused by a vasopressor adjuvant such as epinephrine.

Some believe that this neurological damage is a result of spinal cord ischemia either due to prolonged hypotension during surgery or as a consequence of arterial constriction resulting from the use of epinephrine in the local anesthetics solution [20]. The use of additives in the solution also has been implicated as contributing factors. The pressure of the injected agent may cause nonspecific pressure-related nerve damage.

An immune-mediated mechanism may be possible as suggested by others [4, 16]. In Brummett's study, rat sciatic nerves were harvested at either 24 hours or 14 days after injection and analyzed for perineural inflammation and nerve damage. When compared with the saline control group, the bupivacaine group had significantly higher perineural inflammation scores at 24 hours. Nerves in the bupivacaine and dexmedetomidine group showed less perineural inflammation at 24 hours when compared to the bupivacaine group.

The response to liposomes is well documented and described as a normal foreign body reaction appearing in parallel with liposome deposition. It is generally well accepted that liposomes containing natural phospholipids, triglycerides, and cholesterol should not present any risk of antigenicity, presumably due to their similarities with biological membranes [21]. Natural phospholipids such as phosphatidylcholines with neutral net charge in physiologic conditions are used to construct liposomes that closely resemble biologic membranes. This type of liposomes made of naturally occurring phospholipids is generally considered safe for parenteral use.

Certain types of liposomes may cause extensive tissue damage. Particularly, those composed of lecithin-cholesterol-dicetyl phosphate or lecithin-cholesterol-stearylamine have been reported to cause widespread tissue necrosis, epilepsy, and some deaths due to respiratory failure immediately after injection in mice whereas liposomes composed of phosphatidylcholine cholesterol-phosphatidic acid, or dipalmitoyl phosphatidylcholine only, produced minimal morphological changes and by the sixth day post-injection; the histopathology was limited to the mechanical trauma caused by the injection [22]. Published studies with LipoSpheres containing tristearin and egg phosphatidylcholine in rats have shown no evidence of nerve damage and very little perineural inflammation or foreign body response [23]. Similarly, multilamellar vesicles liposomes made of egg yolk phosphatidylcholine and cholesterol-containing bupivacaine have not been shown to produce histologic lesions on peripheral nerves after either brachial plexus injection [24] or intracerebral administration [25]. Malinovsky et al. has found that the incorporation of bupivacaine into multivesicular liposomes devoid of phosphatidylcholine hydrolysis products or oxidation compounds produce spinal cord histopathologic changes not significantly different from bupivacaine solution after intracisternal administration in rabbits [26].

More recently, drug carriers such as cyclodextrins have shown that the inclusion of bupivacaine 0.5% in hydroxypropyl-[beta]-cyclodextrin in equal amounts produced minimal histological alterations of the rat sciatic nerve 48 hours after intraneural injection [27]. 

During an investigation of the pharmacological activity, cytotoxicity and local effects of ropivacaine 0.125%, 0.25%, and 0.5% concentrations encapsulated into large unilamellar vesicles composed of egg phosphatidylcholine, cholesterol, and alpha-tocopherol (4 : 3 : 0.07, mole %) compared with drug solution showed that there was no morphological tissue changes in the area of injection and sparse inflammatory cells were observed in only one of the animals treated with plain solution or ropivacaine at 0.5% [28].

In sciatic-nerve block experiments in rats, Söderberg et al. [29] showed that after two weeks following perineural injection of various formulations containing 2.0%, 10%, 20%, 60%, or 80% of lidocaine:prilocaine 1 : 1 mixtures in medium chain triglycerides compared to saline, vehicle, 2.0% lidocaine : prilocaine solution and ethanol, pathological changes in the sciatic nerves revealed that the formulations 60% or greater (and ethanol) had neurotoxic effects, that is, axonal swelling and neuronal degeneration, demyelization, and myelin degeneration associated with moderate to marked diffuse inflammation in both the epineural and neuronal tissues. The cellular infiltrate was mainly composed of macrophages, lymphocytes, fibroblasts/fibrocytes, and occasional giant cells.

In a similar study in rats, formulations containing 2%, 4%, 8%, 16%, 32%, or 64% of a mixture of bupivacaine and lidocaine base 4 : 1 in medium-chain triglyceride were evaluated, together with 0.5%, 1.0%, and 2.0% bupivacaine HCl solutions, bupivacaine 4.2% or 7.0% in medium-chain triglyceride, and 20% lidocaine base in a polar lipid vehicle [30]. Histopathologic examination of sciatic nerves by light microscopy revealed slight to moderate signs of neurotoxicity only after administration of the 64% formulation, a week after dosing.

With regard to pathological effects of EXPAREL on peripheral nerves, no remarkable findings were observed using the standard hematoxylin- and eosin-staining method. The brachial plexus sites analyzed for histopathological changes were normal on Day 3 and Day 15. There was no evidence of adverse local reactions even at the highest concentration, 25 mg/mL (30 mg/kg dose). With the exception of granulomatous inflammation, there were no observations of abnormal gross pathology findings at the site of drug administration or elsewhere, and no significant changes in blood chemistry or animal behavior beyond those observed with Bupivacaine solution or saline. It is postulated that macrophages phagocytosed liposome material, as they would any other foreign material in tissues. The increased presence of these cells was therefore not unexpected; the transient local inflammatory response to EXPAREL is a normal foreign body reaction appearing in parallel with liposome deposition. In Boogaerst's study in rabbits, the systemic bupivacaine concentration were lower during the first 10 minutes (*P* < 0.05) and higher after 24 hours (*P* < 0.05) after brachial plexus injection of 2.5 mg bupivacaine in 1 mL of 0.25% multilamellar liposomal bupivacaine made of PC and cholesterol (ratio 4 : 3) compared to bupivacaine solution, while the *C*_max⁡_ was not very different between the two formulations (~0.2 *μ*g/mL) [31].

In our studies, the PK profile displays an initial rise (reflective of unencapsulated drug present in the aqueous phase of EXPAREL) (i.e., outside of the particles) followed by a curve typical of a slow release delivery system (as afforded by the DepoFoam delivery system). In both rabbits and dogs, the peak plasma concentrations of bupivacaine with EXPAREL were significantly attenuated, that is, up to approximately fourfold (9 mg/kg; 106 versus 433 and 402 versus 1490 ng/mL, resp.) compared with equivalent doses of bupivacaine solution (9 mg/kg, *P* < 0.05).

Occasional spurious high *C*_max⁡_ values were observed which accounted for the relatively larger deviation seen with EXPAREL compared with bupivacaine solution. Although the precise nature of the mechanism(s) involved are unknown, there was no compelling evidence that this finding was related to the specific formulation of bupivacaine used (EXPAREL). 

The twofold lower AUC_0–24 h_ with EXPAREL compared to bupivacaine solution reflects a slower absorption in dogs (2860 versus 6020 ng·h/mL, *P* < 0.05 while in rabbits, the AUC_0–24 h_ values were not significantly different (1230 versus 1620 ng·h/mL). Plasma concentrations of bupivacaine were approximately similar or even more prolonged in rabbits (1.6 fold difference in AUC_0–96 h_ *P* < 0.05) consistent with sustained release of EXPAREL. As the toxicity of bupivacaine is known to be generally associated with its *C*_max⁡_, the lower *C*_max⁡_ observed with EXPAREL as compared to bupivacaine solution at the same dose demonstrates potential safety advantages with this liposomal formulation.

The rate of systemic absorption of local anesthetics is dependent upon the total dose and concentration of drug administered, the route of administration, and the vascularity of the administration site. Absorption from the site of injection depends on the blood flow, the more rapid the rate at which plasma concentrations increase and the greater the peak concentration of the drug [7]. It is also possible that the interanimal variability in the PK response may be the consequence of unequal dispersion of the dosing material through the injection site resulting in packed material as well as drug-induced vasodilation so that varied amounts of drug were absorbed.

In summary, a depot formulation with bupivacaine as the active component and DepoFoam lipid carrier was tested after PNB in rabbits and dogs. In the present studies, there were no local signs of toxicity, including no histological evidence for any increase in local reactions or general exacerbations of bupivacaine toxicity after peripheral nerve block. Particularly, there was no evidence of nerve damage at doses up to 30 mg/kg bupivacaine (more than threefold higher doses of EXPAREL versus bupivacaine solution). Bupivacaine did not impact directly on neural tissue, and the findings of granulomatous inflammation were more consistent with a nonspecific foreign—body type reaction most likely mediated by the DepoFoam particles.

Importantly, the DepoFoam delivery system leads to a slower release of the drug allowing a longer duration of action and, from a toxicological standpoint, to a slower uptake into the systemic circulation avoiding high plasma concentrations.

## 5. Conclusions

In conclusion, a single administration of EXPAREL was demonstrated to be safe by peripheral nerve block in rabbits and dogs when tested in comparison with bupivacaine HCl and saline. EXPAREL did not cause overt irritation or local tissue damage even when injected at high dose or concentration around the brachial plexus nerve bundle.

Additional studies are ongoing to further examine the utility of this novel formulation by various routes (e.g., local infiltration, epidural, and intra-articular).

## Figures and Tables

**Table 1:** Injection site microscopic findings in rabbits (combined sexes).

Day 3	Observations	Severity grade	Saline	EXP 9 mg/kg	EXP 18 mg/kg	EXP 30 mg/kg	Bsol 9 mg/kg
Brachial plexus, distal	Hemorrhage	minimal	0	0	1	0	1
		moderate	0	1	1	0	0
	Inflammation, granulomatous	minimal	0	3	1	1	0
	Inflammation, subacute	minimal	0	0	0	0	1

Brachial plexus, middle	Hemorrhage	minimal	0	2	0	1	0
		moderate	0	1	0	0	0
	Inflammation, granulomatous	minimal	0	1	1	3	0
	Inflammation, subacute	minimal	0	0	1	0	0

Brachial plexus, proximal	Hemorrhage	minimal	0	0	1	1	0
		mild	0	1	0	0	0
		moderate	0	1	0	0	0
	Inflammation, granulomatous	minimal	0	0	1	1	0
	Inflammation, subacute	minimal	0	1	1	0	0

Day 15							

Brachial plexus, distal	Hemorrhage	minimal	0	1	1	0	0
		mild	0	0	1	1	0
		moderate	0	0	0	1	0
	Inflammation, subacute	minimal	0	1	0	0	0

Brachial plexus, middle	Fibrosis	minimal	0	0	0	0	1
	Hemorrhage	minimal	0	1	1	0	2
		mild	0	0	0	0	1
	Inflammation, granulomatous	mild	0	0	1	0	0
	Inflammation subacute	minimal	0	1	0	0	0
	Mineralization	minimal	0	0	1	0	0

Brachial plexus, proximal	Hemorrhage	minimal	0	2	0	0	0
		mild	0	1	1	1	0
		moderate	0	0	0	0	1

EXP, EXPAREL (bupivacaine liposome injectable suspension); Bsol, bupivacaine solution.

*Note:* number of animals examined was 4/group on Day 3 or Day 15.

**Table 2:** Injection site findings (Day 3 and Day 15) in dogs (combined sexes).

Day 3	Observations	Severity grade	Saline	EXP 9 mg/kg	EXP 18 mg/kg	EXP 25 mg/kg	Bsol 9 mg/kg
Brachial plexus, distal	Degeneration/regeneration myofibers	mild	0	0	0	0	1
	Hemorrhage, adipose tissue	mild	0	0	0	1	0
		minimal	0	3	1	3	1
	Inflammation, granulomatous, adipose tissue	minimal	0	0	1	1	0
	Inflammation, subacute	minimal	0	0	0	0	1

Brachial plexus, middle	Degeneration/regeneration myofiber	mild	0	0	0	0	1
	Hemorrhage	minimal	0	4	0	1	0
		mild	0	1	2	1	0
		moderate	0	0	0	0	0
	Inflammation, granulomatous	minimal	0	1	0	0	0

Brachial plexus, proximal	Hemorrhage	mild	0	1	2	1	0
	Inflammation, adipose tissue, subacute	mild	0	0	0	1	0

Day 15							

Brachial plexus, distal	Degeneration/regeneration myofibers	mild	0	0	0	0	1
	Hemorrhage	minimal	2	0	1	0	2
Brachial plexus, middle	Cyst	minimal	0	0	1	0	0
	Fibrosis	minimal	0	0	0	0	0
	Hemorrhage	minimal	1	1	1	2	0
		mild	0	0	0	0	1
	Inflammation, granulomatous	minimal	0	0	1	2	0

Brachial plexus, proximal	Degeneration/regeneraton myofibers	minimal	0	0	1	0	1
	Hemorrhage	minimal	0	1	1	0	1
	Inflammation, granulomatous	minimal	0	0	1	1	0
		mild	0	0	1	0	0

Refer to Table 1 footnote.

## Abbreviations

Bsol: Bupivacaine HCl solution
EXP: EXPAREL (bupivacaine liposome injectable suspension using multivesicular DepoFoam technology)
PK: Pharmacokinetics
PNB: Peripheral nerve block.
